# Contribution of Limited Molecular Testing to Low Ehrlichiosis Diagnosis in High Incidence Area, North Carolina, USA

**DOI:** 10.3201/eid3102.240281

**Published:** 2025-02

**Authors:** Alexis M. Siegler, Lauryn Ursery, Dana A. Giandomenico, Melissa B. Miller, Johanna S. Salzer, Alexis M. Barbarin, Carl Williams, Ross M. Boyce

**Affiliations:** University of North Carolina at Chapel Hill, Chapel Hill, North Carolina, USA (A. Siegler, L. Ursery, D.A. Giandomenico, M.B. Miller, R.M. Boyce); Centers for Disease Control and Prevention, Atlanta, Georgia, USA (J.S. Salzer); North Carolina Department of Health and Human Services, Raleigh, North Carolina, USA (A.M. Barbarin, C. Williams)

**Keywords:** Ehrlichiosis, ticks, diagnostic testing, molecular diagnostics, stewardship, tick-borne, bacteria, Ehrlichia, North Carolina, United States, vector-borne infections

## Abstract

Indirect immunofluorescence antibody assays have been the primary method for laboratory diagnosis of ehrlichiosis. Detection of *Ehrlichia* spp. DNA by using PCR is now widely available through commercial laboratories. To prepare for *Ehrlichia* spp. PCR introduction, we assessed ehrlichiosis testing practices, quantified the proportion of samples eligible for PCR testing, and estimated the potential effect of implementing PCR at the University of North Carolina health system in North Carolina, USA, which is in an area with a high-incidence of ehrlichiosis. We found <1% of patient samples underwent PCR testing, even though rates of serodiagnostic algorithm completion (testing of acute and convalescent samples) were low (18.4%). Our findings show a need to educate providers on diagnostic and treatment guidelines for ehrlichiosis and raise awareness of the availability and advantage of PCR testing.

Since 2000, the number of ehrlichiosis cases in the United States reported to the Centers for Disease Control and Prevention (CDC) has increased >10-fold (*1*). Yet, ehrlichiosis is frequently misdiagnosed and underreported because of its relatively nonspecific clinical manifestations (e.g., fever, malaise, headache, myalgia) (*2*). Whereas associated laboratory abnormalities such as thrombocytopenia, hepatic transaminase elevation, and leukopenia may provide further diagnostic clues, similar abnormalities can be seen in other infections. The potential consequences of misdiagnosis are substantial because *Ehrlichia* spp. infection can lead to severe disease. For example, 57% of ehrlichiosis cases reported to CDC during 2008–2012 (1,584 cases) resulted in hospitalization (*3*). Delayed antimicrobial drug treatment is strongly associated with clinical deterioration, characterized by organ failure and death, particularly in children and older adults (*4*,*5*).

Historically, paired acute and convalescent serum samples tested by using an indirect immunofluorescence antibody (IFA) assay that detects IgG against *E. chaffeensis* antigens have been the primary method of laboratory diagnosis (*6*). However, the serodiagnosis of ehrlichiosis is error-prone. IgG may not be detectable early in the course of infection, when most patients seek care (*7*). In addition, a positive acute serologic result may not indicate current infection but rather a prior infection, which may occur in ≈10% of the population in endemic areas (*8*,*9*). Because of the possibility of prior infection, a single acute serologic result cannot be used to confirm a diagnosis, and clinicians must make treatment decisions on the basis of a thorough clinical evaluation, considering a patient’s history, symptoms, and laboratory test results. Providers often wait for the results of the initial IFA assay before beginning antimicrobial drug treatment (*10*), which can lead to disease progression. A second convalescent sample taken 2–10 weeks after the initial acute serologic result is required to confirm the diagnosis of ehrlichiosis and for epidemiologic surveillance data (*11*).

Few patients complete both acute and convalescent testing. For example, in North Carolina, of 105 cases reported in 2020, only 14 (13.3%) were classified as confirmed because of the lack of a paired convalescent result (*12*). Even within a large academic medical center, only 1 in 4 patients tested for ehrlichiosis completed a convalescent test (*13*). Multiple factors may explain the low obtainment of paired serologic results, including the resolution of clinical symptoms because of treatment or self-limited disease, evidence of another cause of illness, or lack of clinician knowledge of testing algorithms.

Molecular approaches that use PCR can detect *Ehrlichia* spp. DNA with high sensitivity and specificity in acute whole blood samples, thereby eliminating the need for convalescent specimens to confirm the diagnosis (*14*). Although treatment for ehrlichiosis must be initiated presumptively, positive *Ehrlichia* PCR results can provide confirmatory evidence of the diagnosis more quickly than paired acute and convalescent samples. Molecular assays can also differentiate between infecting species such as *E*. *chaffeensis*, *E*. *ewingii*, and *E*. *muris eauclairensis*, which can advance our understanding of distinct syndromes associated with each species. However, PCR does have limitations, including increased cost (≈5× that of paired acute and convalescent IFAs at our institution, the University of North Carolina [UNC; Chapel Hill, NC, USA]), uncertainty in the ability to detect *Ehrlichia* sp. DNA after antimicrobial drugs are administered, and the lack of Food and Drug Administration–approved assays.

In preparation for the implementation of a laboratory-developed *Ehrlichia* PCR, we assessed current diagnostic testing practices, quantified the proportion of serologically tested patients that would be eligible for PCR testing, and estimated the potential effect of PCR on the diagnosis and management of ehrlichiosis within the UNC Health System. To accomplish this goal, we conducted a retrospective chart review of all patients tested for *Ehrlichia* over a 12-month period. We hypothesized that serologic testing was poorly targeted, and obtainment of paired specimens (acute and convalescent) would be infrequent, whereas *Ehrlichia* PCR, previously available only through a commercial reference laboratory, was underused.

## Methods

### Data Sources

We obtained diagnostic test results from patients tested for ehrlichiosis as ordered through Epic, the electronic medical record (EMR) system for UNC Health. UNC Health is the largest academic health system in North Carolina, comprising 14 hospitals and ≈500 clinics located throughout the state (*15*). In 2018, UNC Health reported 3.5 million clinical visits, which included nearly 500,000 emergency department visits. Patients were tested for ehrlichiosis by using IFA (Biocell Diagnostics, Inc, http://biocelldx.com) through UNC’s McLendon Clinical Laboratories or by using PCR as a referral or send out test to Mayo Clinical Laboratories (Rochester, MN, USA), which was the institutional provider for *Ehrlichia* PCR testing during March 24, 2022–April 14, 2023 (*16*). Demographic, laboratory, and clinical data associated with each test order were entered into an electronic database (*17*).

### Statistical Analysis

We stratified patients on the basis of 3 criteria: test appropriateness, PCR eligibility, and epidemiologic case classification. We first reviewed symptoms documented in the EMR to identify patients who were appropriately tested, defined as those who met the 2007 Council of State and Territorial Epidemiologists (CSTE) case definition for ehrlichiosis, which was in place at the time the samples were tested (*18*). Although the 2007 CSTE case definition was not intended to determine when to use diagnostics, it offers clear criteria for clinical assessment. Clinical evidence was defined as a subjectively reported or objectively measured fever (temperature >38°C) as documented in the EMR and >1 of the following symptoms: headache, myalgia, anemia (hemoglobin <13.5 g/dL for men, <12.0 g/dL for women), thrombocytopenia (platelets <150/µL), hepatic transaminase elevation (aspartate aminotransferase >33 U/L or alanine aminotransferase >36 U/L), or leukopenia (leukocyte count <4,000 cells/µL) (*18*). We classified patients as eligible for PCR testing if they were prescribed tetracycline antimicrobial drugs the same day as or after initial specimen collection because antimicrobial drugs can remove *Ehrlichia* DNA from patient blood and decrease the sensitivity of PCR testing (*8*). By using the CSTE case classifications, we determined the proportion of cases classified as confirmed, probable, or suspect. A confirmed case demonstrated a 4-fold or greater increase between acute and convalescent IgG titers and consistent clinical evidence of ehrlichiosis. A positive *Ehrlichia* PCR test with consistent clinical evidence was also considered a confirmed case. A probable case did not demonstrate a 4-fold increase but had >1 positive serologic specimen along with clinical evidence of ehrlichiosis. The threshold for a positive serologic test was a 1:64 IgG titer, selected to align with the cutoff used by the CDC to meet laboratory supportive evidence for ehrlichiosis (*11*). A case was classified as suspect if the patient had a positive laboratory test but no clinical information was available to determine if they had relevant symptoms. The number of cases in each category was summarized and shown with relevant proportions.

### Ethical Review

The study was approved by the institutional review board of the University of North Carolina at Chapel Hill (institutional review board no. 21-0356). Because this is a limited dataset under Code of Federal Regulations 45, part 164.514(e), written informed consent or waiver of authorization was not required.

## Results

A total of 945 patient samples were tested for ehrlichiosis during the ≈12-month period observed, 5 of which underwent *Ehrlichia* PCR testing. Among all patients tested, the most frequently recorded symptoms at the time of testing were myalgia (33.9%, n = 320) and headache (31.3%, n = 296) (Table 1). Of those, only 273 (28.9%) were classified as appropriately tested; the most common reasons for exclusion were the absence of documented fever in the EMR (97.9%, n = 658), patient complaint including only 1 symptom (15.8%, n = 106), a lack of any symptoms, or failure to document any symptoms in the EMR (10.4%, n = 70), or a combination. Of the patients who underwent PCR testing, 4 of 5 were not classified as appropriately tested because of the absence of fever. Of note, among the patients excluded, 30.5% reported a tick bite in the 2 weeks before the visit. Most of the appropriately tested patients (93.8%, n = 256) had not received doxycycline at the time of sample collection and therefore would have been eligible for a PCR test (Figure). However, only 1 patient meeting clinical criteria (0.4%) underwent PCR, whereas the remaining had serologic testing performed.

**Table 1:** Clinical symptoms among patients tested for tickborne disease at a visit within the University of North Carolina health system, March 24, 2022–April 14, 2023

Clinical symptoms	No. (%) patients
Meets clinical criteria	273 (28.9)
Fever and headache	151 (55.3)
Fever and myalgia	152 (55.7)
Fever and anemia	92 (33.7)
Fever and thrombocytopenia*	65 (23.8)
Fever and hepatic transaminase elevation†	78 (28.6)
Fever and leukopenia‡	42 (15.4)
Tick bite noticed in previous 2 weeks	77 (28.3)
Does not meet clinical criteria	672 (71.1)
Fever	14 (2.1)
Headache	145 (21.6)
Myalgia	168 (25)
Anemia	50 (7.3)
Thrombocytopenia	28 (4.2)
Hepatic transaminase elevation	47 (7)
Leukopenia	26 (3.9)
Bite noticed in 2 weeks prior	205 (30.5)
No symptoms or bite noticed	69 (10.3)

*Platelets <150,000/μL. †Aspartate aminotransferase >33 U/L or alanine aminotransferase >36 U/L. ‡Leukocytes <4,000 cells/μL.

**Figure:**
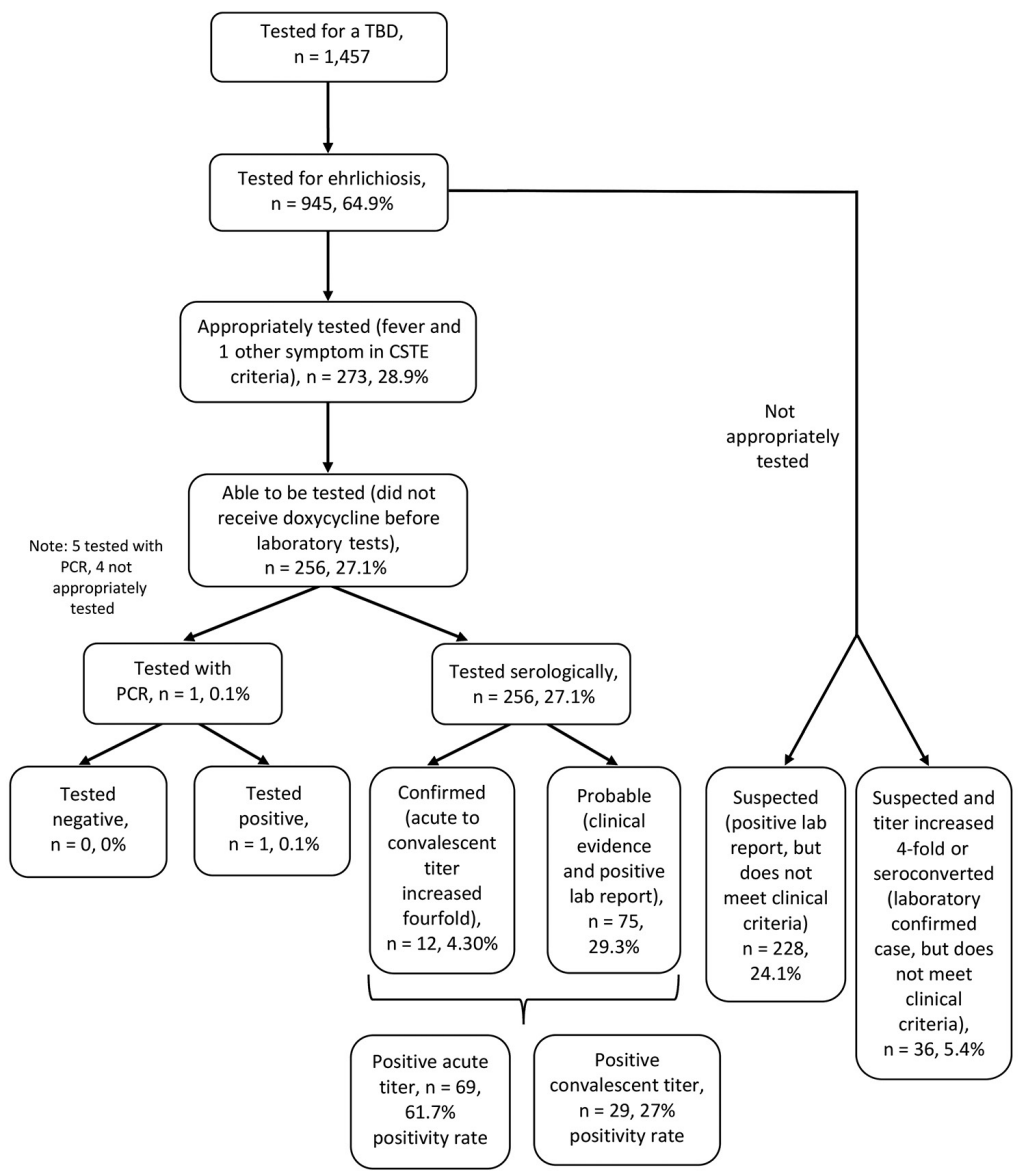
Flowchart describing use of testing to diagnosis ehrlichiosis within the University of North Carolina health system, March 24, 2022–April 14, 2023. CSTE criteria are described in (*18*). CSTE, Council of State and Territorial Epidemiologists; TBD, tickborne disease.

Among the 256 patients who were eligible for PCR, 69 (27.0%) had an acute titer result of >1:64. Only 47 (18.4%) patients completed convalescent testing. Among those who underwent convalescent testing, 6 (12.8%) demonstrated seroconversion and 12 (17.4%) who were positive at acute testing showed a 4-fold or greater titer increase. In addition, 5 (10.6%) reverted from a positive to a negative titer (Table 2). Of note, 106 (38.8%) of 256 patients had a negative acute *Ehrlichia* IgG titer and were started on antimicrobial drugs but did not return for convalescent blood testing. Similarly, 58 (22.7%) of 256 patients had a negative acute titer and neither received antimicrobial drugs nor returned for a convalescent test. Of the 47 patients who completed acute and convalescent *Ehrlichia* IgG testing, the median number of days between tests was 22 (interquartile range 17–32).

**Table 2:** Clinical manifestations and titers of patients confirmed with *Ehrlichia* sp. infection within the University of North Carolina health system, March 24, 2022–April 14, 2023*

Category	Patient identification no.											
	548	549	640	765	935	951	1278	1337	1339	1370	1466	1655
Serologic testing results												
Acute *Ehrlichia* IgG titer	>1:64	1:128	1:128	<1:64	<1:64	>1:64	1:128	<1:64	<1:64	1:128	<1:64	<1:64
Convalescent *Ehrlichia* IgG titer	1:256	1:1,024	1:1,024	1:128	1:1,024	1:1,024	1:512	1:128	1:128	1:512	1:512	1:128

Days between testing	19	30	18	17	19	22	16	29	29	24	21	8
Clinical manifestations												
Fever	Y	Y	Y	Y	Y	Y	Y	Y	Y	Y	Y	Y
Headache	Y	N	Y	Y	Y	Y	N	N	N	N	N	Y
Myalgia	NA	N	N	N	Y	N	Y	Y	Y	N	N	Y
Anemia	Y	Y	Y	Y	Y	Y	N	N	N	Y	Y	N
Thrombocytopenia	N	Y	N	N	Y	Y	Y	N	N	Y	Y	Y
Hepatic transaminase elevation	N	Y	Y	N	N	Y	Y	Y	Y	N	N	Y
Leukopenia	N	N	N	N	N	Y	N	N	N	Y	N	Y

*N, no; NA, not available; Y, yes.

Of the 5 patients who had whole blood samples collected and tested by PCR, 1 test was positive. Attending physicians in the emergency department were responsible for ordering 3 of 5 PCR tests. In all 5 cases, serologic testing for *Ehrlichia* was also conducted. Three of the 5 had an acute but not a convalescent *Ehrlichia* IgG test, whereas 4 of 5 were classified as not appropriately tested because no fever was reported in the EMR (all had >1 other symptom in the CSTE criteria). However, the single positive PCR sample was from a patient whose clinical manifestations included a fever and several other consistent symptoms. The send-out PCR test took an average of 6.2 days to receive a result from the reference laboratory.

Overall, 12 (4.7%) of 256 patients who were eligible for PCR were classified as confirmed ehrlichiosis cases (11 by serology and 1 by PCR and serology); and 75 (29.3%) were classified as probable cases. However, on further review of cases that we defined as not appropriately tested, 228 (33.9%) of 673 patients also had 1 positive *Ehrlichia* spp. test, including 193 (28.7%) who had a positive acute IgG test and 90 (13.4%) who had a convalescent IgG test. Among those who returned for a convalescent test, 35 patients showed seroconversion. Of probable cases, 7 exhibited a 4-fold IgG increase from acute to convalescent titers, raising the total number of laboratory-confirmed cases to 48.

## Discussion

Our study of routine diagnostic testing practices demonstrated that most patients suspected of having ehrlichiosis did not undergo PCR testing when it was available only as a send-out test, despite the multiple advantages of this testing method. Instead, providers continued to rely on serologic testing, even though rates of diagnostic algorithm completion were low (18.4%). Because the lone star tick (which does not transmit *Rickettsia rickettsii*, the causative bacteria of Rocky Mountain spotted fever) is the primary vector in the state (*19*), it is very likely that ehrlichiosis, along with *R. parkeri*, accounts for most symptomatic, tickborne illness episodes (*2*). The limited understanding of the diagnosis and management of ehrlichiosis and the underuse of *Ehrlichia* PCR as a confirmatory assay is particularly concerning. Those issues underscore the need to educate providers at all levels of training and across specialties on the diagnosis and management of ehrlichiosis. In addition, improved uptake of PCR can increase knowledge of the true rate of ehrlichiosis cases in the United States, specifically through a higher rate of confirmation and additional information regarding infecting species.

Several factors may explain the underuse of PCR testing. First, few health facilities have the capability or resources to perform an *Ehrlichia* PCR test in their own laboratories. At the time of this study, *Ehrlichia* PCR was only available at UNC Health as a referral test, with samples sent to Mayo Clinical Laboratories. Many providers may not have been aware that an *Ehrlichia* PCR test was available because it did not appear in the standard order menus. Instead, when ordering an *Ehrlichia* PCR, providers were required to order a generic referral test, which then launched a separate screen where the requested test can be entered as a free text. Ordering the test this way requires knowledge of the *Ehrlichia* PCR, the required sample type, and the receiving laboratory. In contrast, serologic testing, which is performed in-house, can be ordered by a simple search term lookup. This difference in processes might have affected ordering patterns, especially among nonspecialists who are not familiar with the test options. In addition, send-out PCR results still took nearly a week to return to the ordering provider. This timeline might have been insufficient for providers to perceive a benefit to guide treatment decisions, especially when other infections were on the differential diagnosis. Therefore, for optimal implementation, testing likely needs to be performed in-house, and the PCR order should be more prominent and easier to locate within routine test menus.

Whereas empirical antimicrobial drug treatment is recommended when there is a reasonable pretest probability of ehrlichiosis, the relatively fast turnaround of PCR, at least compared with paired serologic testing, offers a timely and objective result to confirm the diagnosis, thereby providing valuable information to both the patient and provider regarding the cause of illness. In our cohort, the number of patients for whom testing was ordered but were not empirically treated with doxycycline is concerning. Because the providers believed there was sufficient evidence to order an ehrlichiosis test, they also should have provided empirical treatment to patients. Without a convalescent blood test, an acute titer result cannot be used to either confirm or rule out ehrlichiosis. Positive PCR results can offer timelier confirmation of ehrlichiosis confirmation and subsequently ensure an appropriate treatment plan. Whereas treatment is ideally prescribed with clinical suspicion, findings show that this is not always the case, and a positive PCR may prompt more immediate action if otherwise not taken. Moreover, because convalescent testing has a much higher positivity rate than acute testing (61.7% vs. 27.0%) but most patients who receive a negative acute IgG titer fail to return for a convalescent titer, there are likely patients with ehrlichiosis who go undetected (*2*). Differing titer values in more than half of paired acute and convalescent samples indicate the convalescent test is clearly needed to interpret serologic results accurately, consistent with current guidelines (*6*). The relatively large proportion of paired titers of the same value implies prior infection and reinforces the importance of paired serologic specimens in areas where baseline seroprevalence is high.

Surprisingly, many patients tested for ehrlichiosis were considered not appropriately tested according to the 2007 CSTE criteria, primarily because of the absence of a subjectively reported fever as documented in the medical record. Yet, the number of laboratory-positive cases and seroconversion rates among patients without clinical evidence was notably higher than that for patients with clinical evidence (36 vs. 12). Those findings suggest that fever is not always a symptom associated with ehrlichiosis. It is also possible that fever is underrecognized because of antipyretic drugs used for headaches and pain control. Largely because of a lack of speciation with serodiagnostic testing, it is possible that infections with non–*E. chaffeensis* species, such as *E. ewingii* and *E. muris eaclairensis*, may exhibit different clinical manifestations than *E. chaffeensis*, which historically is the prototypical pathogen. Previous studies have identified *E. ewingii* in ticks across North Carolina, frequently in higher prevalence than *E. chaffeensis*, which has also been observed in other states (*19*,*20*). Those observations drove changes to the CSTE case definition, which removed fever as a required symptom.

Our study strengths include conduct in an area with high incidence of ehrlichiosis and the use of a robust electronic database. The first limitation of the study is its retrospective nature and that it relied on routine clinical records to determine clinical symptoms associated with manifestation. Second, unknown data, such as the presence of fever not being entered into a patient’s chart, might have resulted in the misclassification of patients. Third, the occurrence of and reliance on single serologic tests has major drawbacks, as described in this article. Fourth, there is potential uncertainty about the true disease state of the patients described in this study. Fifth, the study took place during the COVID-19 pandemic, when care-seeking patterns and diagnostic testing algorithms were disrupted. Finally, we do not have long-term outcomes to assess if participants experienced adverse clinical outcomes because of delayed or lack of antimicrobial drug administration resulting from incomplete testing.

In conclusion, our investigation revealed major underuse of molecular testing for ehrlichiosis in a disease-endemic area, despite the well-established issues associated with serologic testing. Our findings highlight the potential gains that can be made with increased uptake through both provider education and implementation of local testing. Molecular testing could provide information on infecting species, which could help clarify the clinical spectrum, epidemiology, and geographic distribution of different *Ehrlichia* species, and ultimately improve surveillance for this emerging disease and more accurately identify patients at risk of infection.
